# Planning of births and maternal, child health, and nutritional outcomes: recent evidence from India

**DOI:** 10.1016/j.puhe.2018.11.019

**Published:** 2019-04

**Authors:** M.J. Rana, A. Gautam, S. Goli, Uttamacharya, T. Reja, P. Nanda, N. Datta, R. Verma

**Affiliations:** a Centre for the Study of Regional Development, School of Social Sciences, Jawaharlal Nehru University (JNU), New Delhi, India; b International Center for Research on Women (ICRW), New Delhi, India; c Bill & Melinda Gates Foundation (BMGF), India Country Office, New Delhi, India

**Keywords:** Planning of births, Maternal and child health, Nutrition, Family planning, India

## Abstract

**Objectives:**

In an effort to provide recommendation for maximizing synergy between maternal, infant, and young children's nutrition and family planning in India, this study makes a comprehensive assessment of the effects of the planning of births in terms of timing, spacing and limiting childbearing on maternal and child health outcomes.

**Study design:**

This study used the latest National Family Health Survey data of India that is globally known as the Demographic and Health Survey. A robust two-stage systematic random sampling was used for selecting representative samples for measuring demographic and health indicators.

**Methods:**

Maternal and child health outcomes are measured by body mass index (grouped as normal, underweight, and overweight) and anemia for mothers, and stunting, underweight, anemia, and under-five mortality for the children. Logistic regression and Cox proportional hazard models were applied.

**Results:**

Women with a higher number of births and among those with first-order births with fewer than 2 years between marriage and first birth, the risk of being underweight and having anemia was significantly higher compared with their counterparts. In addition, the probability of being underweight and risk of stunting, anemia, and mortality was higher among the children from women with a higher number of births and with fewer than 3 years of spacing between births than that of their counterparts.

**Conclusions:**

The findings from this study support the importance of birth planning in improving maternal, child health, and nutritional outcomes. The proper planning of births could help to achieve the Sustainable Development Goal-3 of good health and well-being for all by 2030 in India, where a significant proportion of women still participate in early marriages, early childbearing, and a large number of births with close spacing.

## Highlights

• Proper planning of births (PoB) leads to better maternal and child health outcomes.
• Health outcomes are better for <3 birth order and >3 years of birth spacing.
• PoB could be more effective in the areas of early marriages and early childbearing.

## Introduction

Maternal, child health, and nutritional outcomes have been key public health issues in developing countries including India. The United Nations has targeted improving maternal, child health, and nutritional outcomes under its Sustainable Development Goal-3 (SDG-3), focusing on the lagging regions by 2030.^1^ The integration of family planning with maternal, newborn, child health, and nutrition services at the program- and policy-level is considered to have natural synergy that benefits women as well as their children. Family planning and maternal, newborn, and child health and nutritional outcomes service integration has shown promising improvement in large variety of health care, processes, and outcomes.^2^ In the global context, the existing evidence indicates that family planning can have a significant influence on achieving key maternal, newborn, and child health and nutritional outcomes.^3^, ^4^ However, a substantial evidence gap continues to persist in the developing countries.

Family planning affects maternal and child health and nutritional outcomes in myriad direct and indirect ways. The integration of family planning with maternal and child health and nutrition services is not a process that occurs in a single episode; instead, it is a continuous process of timing, spacing, and limiting of births. Family planning helps couples plan childbearing regarding timing, spacing, and limiting pregnancy and childbirths.^3^, ^4^ Earlier studies have analyzed the effects of each of these components of the planning of births, such as timing (age at first birth), spacing (birth interval), and limiting (number of children) on maternal, newborn, child health, and nutritional outcomes independently.^5^, ^6^, ^7^, ^8^, ^9^, ^10^ The timing of the first birth is assessed based on the timing of marriage, timing of the first birth after marriage, and the gap between the date of the marriage and the first birth. The timing of the first birth is related to maternal, newborn, child health, and nutritional outcomes.^11^ The timing of the first birth at a maternal adolescent age adversely affects the women's health because they are in a critical period of physical growth that is hindered by pregnancy and childbearing.^12^, ^13^, ^14^, ^15^, ^16^, ^17^ However, even if marriage takes place at an early age, postponement of childbearing until women become physically and psychologically capable will result in better pregnancy and delivery outcomes.^12^, ^13^, ^14^, ^15^, ^16^, ^17^ The use of family planning helps in the postponement of childbearing to optimal ages.^17^

On the other hand, a shorter birth spacing and a higher number of births due to repeated childbearing as a consequence of poor acceptance of family planning, higher unmet need for family planning, and more unintended births in turn lead to poor maternal, newborn, and child health and nutritional outcomes.^7^, ^8^, ^9^, ^18^, ^19^ Insufficient birth spacing and repeated childbearing cause the recurrent loss of macronutrients and micronutrients from the women's body during the pregnancy, delivery, and breastfeeding.^15^, ^17^, ^20^ Such unplanned childbearing elevates the risk of intrauterine growth restriction (IUGR), low birth weight (LBW), premature birth, and small birth size. The poor pregnancy and delivery outcomes for the baby make them vulnerable to reduced physical growth and add to the risk of mortality during their childhood.^2^, ^15^, ^20^

On the other hand, studies that used direct measures and indirect proxies of family planning such as unintended births, in particular when based on cross-sectional data from demographic and health surveys (DHSs),^21^, ^22^ lack a comprehensive outline of empirical evidence on the pathways of the influence of family planning on maternal, newborn, child health and on nutritional outcomes. Moreover, unintended births are not only just a result of not having access to family planning or the failure of it but also due to other social or cultural reasons.^23^ Recent evidence suggests that the rate of unintended births has been falling and the fertility is declining in India.^24^, ^25^, ^26^, ^27^ Therefore, an unintended birth is not a good proxy to predict family planning. Furthermore, limitations related to family planning questions in DHS data do not allow the direct linking of family planning to maternal, child health, and nutritional outcomes.^4^

Although, progress in age at first birth, birth order, and birth interval helps to achieve favorable for maternal, child health, and nutritional outcomes, but the best outcomes will be possible with the right combinations of all three components comprising a comprehensive framework of planning of births are not identified in the previous studies.^5^, ^6^, ^7^, ^8^, ^9^, ^10^ For instance, many states of India overdrive to achieve replacement level fertility through female sterilizations which have led to certainly rapid decline in fertility, but at the same time, there is only moderate progress in age at first birth and hardly any improvement in birth interval.^24^, ^25^, ^26^, ^27^, ^28^ In a paradoxical situation of declining fertility with stalling, unmet need for family planning demands a deeper understanding into planning of births in India. Therefore, this article advances an argument for comprehensive strategy of planning of births (through appropriate timing, spacing, and limiting of births) rather than individual components in a context where the levels of contraceptive use is declining as evident from the recent National Family and Health Survey (NFHS).^26^, ^27^ A few recent studies have used the intersectional axes of the continuum process of planning of births (as a proxy of family planning outcomes) to predict differential maternal and child nutritional outcomes among women who adopted better family planning compared with that of their counterparts. The detailed theoretical framework showing linkages between family planning and maternal and health nutritional outcomes has been discussed elsewhere.^3^, ^4^ Using the framework prepared in the context of South Asia, this study aims to provide comprehensive empirical evidence showing the effects of the intersectional axes of the planning of births on the select key maternal, child health and nutritional outcomes using a recent nationally representative large-scale database available in India.

## Methods

The data for the current study have been taken from the fourth round of the NFHS conducted during 2015–16.

### Sampling design and sample size

The data consisting of essential health and family welfare indicators have been collected by interviewing 699,686 women. The women were selected using two-stage systematic random sampling. However, in the analyses of this study, only those women who had at least one child were included. Thus, the final sample accounts for 437,501 and 461,141 for body mass index (BMI) and anemia, respectively. The final sample size in cases of child undernutrition and anemia analyses account for 223,011 and 207,594, respectively. The childhood mortality analysis was based on a total sample of 526,868 live births delivered in the 10 years preceding the date of the survey.

### Outcome variables

We have considered maternal, child nutritional status and childhood mortality as outcome indicators for the study. BMI and anemia have been considered as indicators of maternal health and nutritional outcomes, while child health outcomes have been measured by stunting, underweight, anemia, and mortality. Maternal BMI has been categorized into three groups as per WHO guidelines viz. undernourished (<18.5 kg/m^2^), normal (18.5–24.9 kg/m^2^), and obese (≥25 kg/m^2^). The children with less than −2 standard deviations of height-for-age and weight-for-age have been grouped into stunted and underweight, respectively. Any anemia, including severe and less severe, has been considered for both the women and their children. The under-five mortality rates have been estimated indirectly from the survival rates of the life tables of last 10 years of birth history.

### Predictors

The predictor variable, the intersectional axes of the planning of births, has been created using the continuum process of timing, spacing, and limiting of births, namely, the interval between marriage and first birth (IBMFB), the interval between a birth and a subsequent birth (IBBSB), and birth order. The birth order has been categorized into 1, 2, 3, and > 3. The IBMFB has been grouped into <2 years, 2–3 years, and >3 years for the first birth order; whereas, for birth order >1, the IBBSB has been divided into <3 years and ≥3 years. Altogether, nine intersectional axes of the planning of births have been created. The socio-economic, demographic, and other associated confounders (region) have been considered for the multivariate analyses. The region is divided into four categories: Uttar Pradesh, Bihar, the Empowered Action Groups (EAG) states, and others (the remaining states of India).

### Statistical analyses

We used both bivariate and multivariate statistical analyses to establish the association between the planning of births and maternal, newborn, and child health and nutritional outcomes. The multinomial logistic regression and multiple classification analysis conversion model was applied to estimate the adjusted association between the planning of births and maternal BMI, while separate binary logistic regression models were used to assess the effect of the planning of births on child stunting, underweight, and women and child's anemia. As a postestimation of the regression models, the predicted probabilities have been estimated and converted into percentages for ease of interpretation. Furthermore, the assessment of the effects of the planning of births on under-five mortality was carried out using the Cox-proportional hazard regression model.

## Results

### Characteristics of the participants

The univariate sample distribution by outcome and predictor variables has been displayed in Table 1 for the women and children separately. The estimation of nutritional status and anemia levels in women suggests that approximately 18% are underweight, while 52% are anemic. Approximately, 38%, 35%, and 58% of the children are stunted, underweight, and anemic, respectively. The sample distribution for women varies from a minimum of 5% to a maximum of 19% in different axes of the planning of births (Table 1). The percentage of the total sample for the children for different intersectional axes of the planning of births ranges from a low of 7% to a high of 18% (Table 1). Overall, across the sample, the percentage of women having shorter (<3 years) birth spacing is higher than the percentage of those who have had the longer spacing (>3 years).

**Table 1:** Descriptive statistics of the outcome variables and predictor of this study, 2015–16.

Variables	Body mass index		Women anemia		Child undernutrition		Child anemia		Under-five mortality	
	*n*	% (95% CI)	N	% (95% CI)	*n*	% (95% CI)	*n*	% (95% CI)	*n*	% (95% CI)
*BMI*										
Underweight	78,880	18.2 (18.1–18.3)	–	–	–	–	–	–	–	–
Normal	2,56,214	58.7 (58.5–58.8)	–	–	–	–	–	–	–	–
Obese	1,02,407	23.1 (23.0–23.3)	–	–	–	–	–	–	–	–
Women anemia	–	–	2,40,773	52.2 (52.1–52.4)	–	–	–	–	–	–
Child stunting	–	–	–	–	86,239	38.1 (37.9–38.3)	–	–	–	–
Child underweight	–	–	–	–	77,723	34.5 (34.3–34.7)	–	–	–	–
Child anemia	–	–	–	–	–	–	1,19,560	57.6 (57.4–57.8)	–	–
*Planning of births*										
Order 1 & <2 years of IBMFB	32,512	7.6 (7.6–7.7)	38,184	8.3 (8.2–8.4)	37,926	16.2 (16.1–16.4)	34,638	16.7 (16.5–16.8)	82,663	15.7 (15.6–15.8)
Order 1 & 2–3 years of IBMFB	19,764	4.7 (4.6–4.8)	22,970	5.0 (4.9–5.0)	22,668	9.7 (9.6–9.8)	21,097	10.2 (10.0–10.3)	49,936	9.5 (9.4–9.6)
Order 1 & >3 years of IBMFB	21,950	5.3 (5.2–5.3)	24,688	5.4 (5.3–5.4)	21,001	9.1 (9.0–9.2)	19,624	9.5 (9.3–9.6)	49,103	9.3 (9.2–9.4)
Order 2 & <3 years of IBBSB	82,169	18.8 (18.7–19.0)	86,361	18.7 (18.6–18.8)	40,845	18.3 (18.2–18.5)	38,224	18.4 (18.2–18.6)	94,918	18.0 (17.9–18.1)
Order 2 & >3 years of IBBSB	65,262	14.7 (14.6–14.8)	67,269	14.6 (14.5–14.7)	28,405	13.4 (13.2–13.5)	26,169	12.6 (12.5–12.7)	61,216	11.6 (11.5–11.7)
Order 3 & <3 years of IBBSB	59,675	13.6 (13.5–13.7)	61,603	13.4 (13.3–13.5)	20,662	9.3 (9.2–9.5)	19,476	9.4 (9.3–9.5)	53,563	10.2 (10.1–10.2)
Order 3 & >3 years of IBBSB	42,541	9.5 (9.5–9.6)	43,535	9.4 (9.4–9.5)	15,560	7.3 (7.2–7.5)	14,414	6.9 (6.8–7.1)	35,253	6.7 (6.6–6.8)
Order >3 & <3 years of IBBSB	70,349	16.0 (15.9–16.1)	72,224	15.7 (15.6–15.8)	20,757	9.5 (9.4–9.6)	19,673	9.5 (9.4–9.6)	63,044	12.0 (11.9–12.1)
Order >3 & >3 years of IBBSB	43,279	9.7 (9.6–9.8)	44,307	9.6 (9.5–9.7)	15,187	7.2 (7.0–7.3)	14,279	6.9 (6.8–7.0)	37,172	7.1 (7.0–7.1)
*Total*	4,37,501	100	4,61,141	100	2,23,011	100	2,07,594	100	5,26,868	100

*n*, unweighted sample size; IBMFB, interval between marriage and first birth; IBBSB, interval between birth and subsequent birth; CI, confidence interval; BMI, body mass index.

Upper and lower limit of confidence interval have been shown in the parentheses.

### Women's health outcomes

Table 2 shows the percentage of underweight, normal, and obese mothers by the axes of the planning of births adjusted for the other socio-economic and demographic confounders. As the interest of this study is only the underweight women, hereafter, normal and obese categories of women will not be discussed. The results suggest that the probability of being underweight is higher among women with >3 births and <3 years IBBSB (20.7%, *P*<0.01) and >3 years of IBBSB (22.3%, *P* < 0.01) compared with that for women with one birth and 2–3 years of IBMFB (18.9%, *P* < 0.10), three births, and <3 years of IBBSB (18.1%, *P* < 0.10) and 3 births and >3 years of IBBSB (17.4%, *P* < 0.01). The EAG states (23.3%, *P* < 0.01), Uttar Pradesh (19.1%, *P* < 0.01), and Bihar (26.6%, *P* < 0.01) have a higher number of underweight mothers compared with that of the rest of India (14.3%). Along with the planning of births and place of residence (region), other factors such as demographic and socio-economic variables were also significantly associated with women being underweight.

**Table 2:** Results from multivariate regression analysis: adjusted percentages of underweight, normal, obese and anemic women by the selected factors in India, 2015–16 [% (95% CI)].

Variables	Body mass index^a^			Anemia^b^
	Underweight	Normal®	Obese	
*Planning of births*				
Order 1 & <2 years of IBMFB®	19.4 (19.3–19.6)	58.4 (58.4–58.5)	22.2 (22.0–22.3)	51.8 (51.7–51.8)
Order 1 & 2–3 years of IBMFB	18.9 (18.7–19.1)*	58.9 (58.8–59.0)	22.2 (22.0–22.4)	52.3 (52.2–52.4)
Order 1 & >3 years of IBMFB	18.9 (18.8–19.1)	55.3 (55.2–55.4)	25.7 (25.5–26.0)***	52.4 (52.3–52.5)*
Order 2 & <3 years of IBBSB	16.8 (16.7–16.9)	55.8 (55.7–55.8)	27.4 (27.3–27.5)**	53.4 (53.3–53.4)***
Order 2 & >3 years of IBBSB	14.1 (14.0–14.2)	54.0 (54.0–54.1)	31.9 (31.7–32.0)*	52.8 (52.8–52.8)***
Order 3 & <3 years of IBBSB	18.1 (18.0–18.2)*	56.7 (56.6–56.7)	25.2 (25.1–25.4)	54.4 (54.4–54.5)***
Order 3 & >3 years of IBBSB	17.4 (17.3–17.5)***	55.0 (54.9–55.1)	27.6 (27.4–27.8)	54.9 (54.8–54.9)***
Order >3 & <3 years of IBBSB	20.7 (20.6–20.8)***	57.4 (57.3–57.4)	21.9 (21.8–22.0)	54.6 (54.6–54.7)***
Order >3 & >3 years of IBBSB	22.3 (22.1–22.4)***	56.9 (56.8–56.9)	20.9 (20.7–21.0)**	55.5 (55.4–55.5)***
*Age at marriage in years*				
<15®	19.3 (19.2–19.4)***	56.5 (56.5–56.6)	24.2 (24.0–24.3)	54.5 (54.5–54.6)
15–19	19.7 (19.6–19.7)***	56.6 (56.6–56.7)	23.7 (23.7–23.8)***	54.3 (54.3–54.3)**
20–24	15.2 (15.1–15.2)***	55.8 (55.7–55.8)	29.1 (29.0–29.2)***	52.2 (52.2–52.2)***
25–29	11.9 (11.8–12.1)***	53.6 (53.4–53.7)	34.5 (34.3–34.7)***	50.0 (49.9–50.1)
30+	11.8 (11.4–12.1)*	55.7 (55.5–56.0)	32.5 (31.9–33.1)***	47.6 (47.4–47.8)
Not reported	18.0 (17.8–18.1)***	55.4 (55.3–55.5)	26.6 (26.4–26.8)***	56.5 (56.4–56.5)***
*Current age in years*				
15–19®	36.8 (36.6–37.1)***	57.0 (56.8–57.1)	6.2 (6.1–6.3)	60.7 (60.6–60.8)***
20–24	29.9 (29.8–30.0)***	59.5 (59.5–59.6)	10.6 (10.5–10.6)***	57.2 (57.2–57.3)***
25–29	21.9 (21.8–21.9)***	59.8 (59.8–59.8)	18.3 (18.3–18.4)***	53.8 (53.7–53.8)***
30–34	17.0 (17.0–17.1)***	57.2 (57.1–57.2)	25.8 (25.7–25.9)***	52.1 (52.1–52.1)***
35–39	14.4 (14.3–14.4)***	55.9 (55.9–56.0)	29.7 (29.6–29.8)***	53.1 (53.0–53.1)***
40–44	14.0 (13.9–14.0)***	52.7 (52.7–52.8)	33.3 (33.2–33.4)***	53.5 (53.5–53.6)***
45–49	13.3 (13.2–13.4)***	52.1 (52.0–52.2)	34.6 (34.5–34.7)***	52.7 (52.6–52.7)***
*Place of residence*				
Urban®	9.5 (9.5–9.6)	50.9 (50.8–50.9)	39.6 (39.5–39.7)	51.3 (51.2–51.3)
Rural	22.3 (22.2–22.3)***	58.9 (58.8–58.9)	18.8 (18.8–18.9)***	54.8 (54.8–54.8)**
*Religion*				
Hindu®	18.8 (18.8–18.9)	56.7 (56.7–56.7)	24.5 (24.4–24.5)	54.2 (54.2–54.2)
Muslim	15.7 (15.6–15.7)***	53.8 (53.7–53.9)	30.5 (30.4–30.7)***	51.2 (51.2–51.3)***
Christian	10.6 (10.5–10.8)***	55.1 (54.9–55.3)	34.2 (33.9–34.6)***	49.0 (48.9–49.1)***
Others	12.5 (12.3–12.7)***	54.1 (54.0–54.3)	33.4 (33.1–33.7)**	53.3 (53.2–53.3)
*Caste*				
Others®	12.7 (12.6–12.8)	53.4 (53.3–53.4)	33.9 (33.8–34.0)	50.2 (50.2–50.2)
SC	20.5 (20.4–20.5)*	58.0 (58.0–58.1)	21.5 (21.4–21.6)***	56.2 (56.2–56.3)***
ST	29.2 (29.1–29.3)***	58.7 (58.6–58.7)	12.1 (12.0–12.2)***	60.3 (60.3–60.4)***
OBC	17.5 (17.5–17.6)**	56.3 (56.2–56.3)	26.2 (26.1–26.3)***	52.9 (52.9–52.9)***
Do not know/not reported	15.7 (15.6–15.8)**	56.1 (56.0–56.3)	28.2 (27.9–28.4)**	52.2 (52.2–52.3)*
*Women's education*				
Illiterate®	23.8 (23.8–23.9)	58.5 (58.4–58.5)	17.7 (17.7–17.8)	56.3 (56.3–56.4)
Primary	19.3 (19.2–19.4)***	56.4 (56.3–56.4)	24.3 (24.2–24.5)***	54.5 (54.5–54.6)**
Secondary	14.7 (14.6–14.7)***	54.7 (54.6–54.7)	30.6 (30.6–30.7)***	52.2 (52.2–52.2)***
Higher	7.5 (7.4–7.5)***	53.5 (53.4–53.6)	39.0 (38.9–39.2)***	47.9 (47.8–47.9)***
*Partner's education*				
Illiterate®	24.5 (24.4–24.7)	57.9 (57.8–58.0)	17.6 (17.4–17.8)	57.9 (57.8–58.0)
Primary	20.8 (20.6–21.0)	56.8 (56.7–56.9)	22.4 (22.1–22.7)	55.1 (55.0–55.2)
Secondary	16.2 (16.1–16.3)	54.3 (54.2–54.4)	29.5 (29.3–29.7)**	53.3 (53.2–53.3)
Higher	8.9 (8.8–9.1)	52.3 (52.1–52.5)	38.8 (38.4–39.1)	48.9 (48.8–49.0)*
Not reported	18.1 (18.1–18.1)*	56.4 (56.4–56.4)	25.5 (25.4–25.5)	53.6 (53.6–53.6)
*Occupation*				
Not working®	16.5 (16.4–16.6)	54.2 (54.1–54.2)	29.4 (29.2–29.5)	53.4 (53.4–53.4)
White collar worker	7.5 (7.2–7.7)***	52.1 (51.8–52.5)	40.4 (39.9–40.9)	49.6 (49.4–49.7)
Agricultural worker	25.0 (24.8–25.2)***	60.4 (60.3–60.5)	14.6 (14.4–14.7)**	57.3 (57.2–57.3)
Service sector/manual worker	17.3 (17.0–17.5)	54.5 (54.3–54.7)	28.2 (27.9–28.6)*	53.7 (53.6–53.8)
Do not know/not reported	18.1 (18.1–18.1)	56.4 (56.4–56.4)	25.5 (25.4–25.6)	53.6 (53.6–53.6)
*Wealth status*				
Poorest®	33.2 (33.2–33.3)	59.6 (59.5–59.6)	7.2 (7.2–7.2)	59.1 (59.1–59.2)
Poorer	25.2 (25.1–25.2)***	60.7 (60.7–60.7)	14.1 (14.1–14.2)***	55.8 (55.7–55.8)***
Middle	17.3 (17.2–17.3)***	59.2 (59.2–59.2)	23.6 (23.5–23.6)***	53.7 (53.7–53.7)***
Richer	10.9 (10.9–11.0)***	53.7 (53.6–53.7)	35.4 (35.3–35.5)***	51.4 (51.4–51.5)***
Richest	5.6 (5.6–5.6)***	48.3 (48.2–48.4)	46.1 (46.1–46.2)***	48.6 (48.6–48.7)***
*Exposure to mass media*				
No®	24.3 (24.3–24.4)	58.2 (58.2–58.3)	17.4 (17.4–17.5)	55.9 (55.8–55.9)
Partial	14.8 (14.8–14.8)***	55.3 (55.3–55.4)	29.9 (29.8–29.9)***	52.5 (52.5–52.5)
Full	11.3 (11.2–11.3)***	53.1 (53.0–53.2)	35.6 (35.5–35.7)***	51.4 (51.4–51.5)
*Regions*				
Others®	14.3 (14.3–14.3)	54.0 (53.9–54.0)	31.7 (31.7–31.8)	53.8 (53.7–53.8)
EAG	23.3 (23.2–23.3)***	59.4 (59.4–59.5)	17.3 (17.2–17.4)***	51.3 (51.3–51.3)***
Uttar Pradesh	19.1 (19.0–19.2)***	58.1 (58.1–58.2)	22.8 (22.6–22.9)***	52.9 (52.8–52.9)***
Bihar	26.6 (26.5–26.7)***	58.7 (58.6–58.8)	14.7 (14.6–14.8)***	60.8 (60.8–60.8)***
*Total*	18.0 (18.0–18.1)	56.2 (56.2–56.2)	25.8 (25.7–25.8)	53.7 (53.6–53.7)
*Number of observations*		4,37,501		4,61,141
*Log pseudo likelihood*		−394224		−319963
*Wald chi*^*2*^		30295.36***		2840.17***

CI, confidence interval; IBMFB, interval between marriage and first birth; IBBSB, interval between birth and subsequent birth; MCA, multiple classification analysis; EAG, Empowered Action Group.

Upper and lower limit of confidence interval have been shown in the parentheses; Estimates are weighted with national women weight; *P < 0.10, **P < 0.05, ***P < 0.01. ® stands for reference category of the variable.

a Estimates based on multinomial regression and MCA conversional model.

b Estimates based on logistic regression model and MCA conversional model.

Table 2 presents the percentage of women with anemia by the planning of births after controlling for the other socio-economic and demographic predictors reported in the previous studies. The results show that the likelihood of anemia is significantly higher among the women with two births and <3 years of IBBSB (53.4%, *P* < 0.01), three births and <3 years of IBBSB (54.4%, *P* < 0.01), three births and >3 years of IBBSB (54.9%, *P*<0.01), >3 births and <3 years of IBBSB (54.6%, *P*<0.01) and >3 births and >3 years of IBBSB (55.5%, *P* < 0.01) compared with that for one birth and >3 years of IBMFB (52.4%, *P* < 0.10). The risk of anemia was lower in the EAG states (51.3%, *P* < 0.01) and Uttar Pradesh (52.9%, *P* < 0.01) but was substantially higher in Bihar (60.8%, *P* < 0.01) than that in the other states of India (53.8%). Similar to previous studies, the socio-economic and demographic factors reported in Table 3 have also emerged as significant correlates of women's anemia, excluding the occupational status of the mothers.

**Table 3:** Results from multivariate regression analysis: adjusted percentages of childhood undernutrition, anemia, and hazard ratios of under-five mortality by selected factors in India, 2015–16.

Variables	Undernutrition^a^		Anemia^a^ [% (95% CI)]	Mortality^b^ [hazard ratio (95% CI)]
	Stunting [% (95% CI)]	Underweight [% (95% CI)]		
*Planning of births*				
Order 1 & <2 years of IBMFB®	32.5 (32.0–33.0)	30.7 (30.3–31.2)	54.1 (53.6–54.7)	–
Order 1 & 2–3 years of IBMFB	33.0 (32.4–33.6)*	32.1 (31.5–32.7)	56.1 (55.4–56.7)*	0.81 (0.74–0.89)***
Order 1 & >3 years of IBMFB	35.7 (35.0–36.3)	33.5 (32.9–34.1)	57.5 (56.8–58.2)***	0.88 (0.80–0.96)***
Order 2 & <3 years of IBBSB	41.1 (40.6–41.6)***	38.1 (37.7–38.6)***	60.2 (59.7–60.7)***	0.96 (0.89–1.04)
Order 2 & >3 years of IBBSB	32.2 (31.6–32.7)	30.1 (29.6–30.6)	55.8 (55.2–56.4)**	0.61 (0.55–0.67)***
Order 3 & <3 years of IBBSB	46.8 (46.1–47.5)***	43.0 (42.3–43.7)***	63.5 (62.8–64.2)***	1.11 (1.02–1.21)**
Order 3 & >3 years of IBBSB	37.9 (37.1–38.7)	35.1 (34.3–35.9)	59.4 (58.6–60.3)***	0.62 (0.56–0.69)***
Order >3 & <3 years of IBBSB	53.1 (52.4–53.8)***	48.4 (47.6–49.1)***	64.9 (64.2–65.7)***	1.37 (1.25–1.49)***
Order >3 & >3 years of IBBSB	47.3 (46.5–48.2)***	43.5 (42.6–44.4)***	62.6 (61.7–63.4)***	0.72 (0.65–0.80)***
*Age at marriage in years*				
<15®	46.4 (45.6–47.1)	42.4 (41.7–43.1)	60.3 (59.6–61.0)	–
15–19	41.2 (41.0–41.5)	38.4 (38.2–38.7)**	60.0 (59.8–60.3)	0.97 (0.91–1.02)
20–24	33.8 (33.4–34.2)	31.7 (31.4–32.1)	57.1 (56.7–57.5)*	0.89 (0.83–0.95)***
25–29	27.4 (26.6–28.3)**	25.5 (24.8–26.3)	51.1 (50.2–52.1)	0.89 (0.79–0.99)**
30+	25.8 (23.7–28.1)***	27.5 (25.3–29.8)	51.2 (48.6–53.8)	0.94 (0.76–1.16)
Not reported	46.9 (44.2–49.5)	44.7 (42.1–47.4)**	58.2 (55.5–60.7)	1.29 (1.14–1.47)***
*Current age in years*				
15–19®	32.8 (31.6–34.0)	33.5 (32.3–34.7)	64.8 (63.4–66.2)	–
20–24	37.7 (37.3–38.1)	35.7 (35.4–36.1)	61.5 (61.2–61.9)	0.63 (0.53–0.76)***
25–29	38.4 (38.0–38.7)	35.6 (35.2–35.9)	57.9 (57.5–58.2)	0.63 (0.53–0.76)***
30–34	39.3 (38.8–39.8)*	36.2 (35.7–36.6)	56.1 (55.6–56.6)*	0.62 (0.51–0.74)***
35–39	42.8 (41.9–43.6)	38.9 (38.1–39.7)	55.3 (54.4–56.1)***	0.61 (0.50–0.74)***
40–44	48.6 (46.9–50.2)	45.0 (43.4–46.7)	58.9 (57.2–60.5)	0.71 (0.58–0.87)***
45–49	53.2 (50.1–56.3)	45.6 (42.5–48.8)	57.9 (54.8–60.9)**	0.76 (0.61–0.94)**
*Mother's BMI*				
Normal®	37.4 (37.1–37.7)	33.8 (33.5–34.0)	–	–
Underweight	45.9 (45.5–46.4)***	47.9 (47.5–48.4)***	–	1.07 (1.02–1.12)***
Obese	26.7 (26.2–27.3)***	21.7 (21.2–22.1)***	–	0.96 (0.90–1.02)
Not reported	43.6 (43.0–44.2)***	39.5 (38.9–40.2)***	–	1.76 (1.66–1.87)***
*Mother's anemia*				
Not anemic®	–	–	50.6 (50.3–51.0)	–
Anemic	–	–	64.8 (64.5–65.1)***	–
Not reported	–	–	54.3 (50.7–57.8)*	–
*Place of residence*				
Urban®	31.3 (30.9–31.7)	29.6 (29.2–29.9)	56.1 (55.7–56.6)	–
Rural	41.5 (41.3–41.8)***	38.6 (38.3–38.8)***	59.6 (59.4–59.9)***	0.99 (0.93–1.04)
*Religion*				
Hindu®	38.8 (38.6–39.0)	36.6 (36.4–36.9)	58.9 (58.6–59.1)	–
Muslim	40.1 (39.6–40.6)**	35.2 (34.7–35.7)*	59.3 (58.7–59.8)	0.99 (0.93–1.05)
Christian	30.1 (28.7–31.5)***	27.3 (26.0–28.7)***	45.9 (44.4–47.5)***	1.43 (1.31–1.55)***
Others	33.4 (32.2–34.6)	31.5 (30.4–32.7)	58.8 (57.5–60.1)**	1.04 (0.93–1.16)
*Caste*				
Others®	30.9 (30.5–31.4)	28.9 (28.4–29.3)	54.7 (54.2–55.2)	–
SC	43.1 (42.6–43.5)***	39.6 (39.1–40.0)***	60.8 (60.3–61.2)***	1.19 (1.11–1.27)***
ST	44.2 (43.6–44.9)***	45.4 (44.8–46.1)***	63.8 (63.1–64.5)***	1.26 (1.17–1.36)***
OBC	39.0 (38.7–39.3)***	35.9 (35.6–36.2)***	58.7 (58.4–59.0)***	1.03 (0.97–1.10)
Do not know/not reported	34.8 (33.8–35.8)	30.2 (29.2–31.1)*	53.1 (52.1–54.2)***	1.02 (0.91–1.14)
*Mother's education*				
Illiterate	51.1 (50.7–51.5)	47.2 (46.8–47.6)	65.0 (64.6–65.4)	–
Primary	43.8 (43.2–44.3)***	40.3 (39.8–40.9)***	60.7 (60.1–61.2)***	0.93 (0.88–0.98)***
Secondary	33.0 (32.7–33.3)***	31.4 (31.1–31.7)***	55.8 (55.5–56.1)***	0.72 (0.69–0.76)***
Higher	21.1 (20.6–21.6)***	19.1 (18.6–19.6)***	49.6 (48.9–50.3)***	0.49 (0.43–0.57)***
*Father's education*				
Illiterate®	51.4 (50.2–52.6)	47.5 (46.3–48.8)	64.5 (63.2–65.7)	–
Primary	43.4 (42.1–44.8)	41.2 (39.8–42.5)	61.9 (60.5–63.2)	0.90 (0.78–1.02)*
Secondary	35.9 (35.2–36.6)***	33.2 (32.6–33.9)**	57.1 (56.4–57.8)	0.80 (0.71–0.89)***
Higher	23.9 (22.8–25.1)***	22.4 (21.3–23.6)***	51.1 (49.7–52.6)	0.60 (0.47–0.76)***
Not reported	38.8 (38.6–39.0)	36.2 (36.0–36.5)	58.7 (58.5–59.0)	0.81 (0.56–1.17)
*Mother's occupation*				
Not working®	36.7 (36.1–37.2)	34.0 (33.5–34.6)	58.1 (57.5–58.7)	–
White collar worker	27.5 (24.8–30.4)	23.7 (21.1–26.4)	49.5 (46.4–52.7)	0.94 (0.69–1.27)
Agricultural worker	46.3 (44.8–47.8)	44.2 (42.8–45.7)	61.5 (60.1–63.0)	0.86 (0.77–0.97)**
Service sector/manual worker	42.6 (40.7–44.6)	39.6 (37.7–41.5)	59.1 (57.1–61.1)	1.02 (0.87–1.18)
Do not know/not reported	38.8 (38.6–39.0)	36.2 (36.0–36.5)	58.7 (58.5–59.0)	1.06 (0.73–1.53)
*Wealth status*				
Poorest®	51.8 (51.4–52.2)	49.0 (48.6–49.4)	64.2 (63.7–64.6)	–
Poorer	43.8 (43.4–44.3)***	40.8 (40.4–41.2)***	59.9 (59.4–60.3)***	0.86 (0.82–0.90)***
Middle	36.8 (36.3–37.2)***	33.7 (33.3–34.2)***	59.0 (58.6–59.5)	0.79 (0.74–0.84)***
Richer	29.5 (29.0–29.9)***	27.7 (27.3–28.1)***	54.5 (54.0–55.0)***	0.72 (0.67–0.78)***
Richest	22.5 (22.0–23.0)***	20.4 (20.0–20.9)***	51.9 (51.3–52.5)***	0.59 (0.53–0.65)***
*Exposure to mass media*				
No®	45.7 (45.4–46.1)	42.6 (42.3–42.9)	61.8 (61.5–62.2)	–
Partial	34.7 (34.4–35.0)**	32.5 (32.2–32.8)*	56.9 (56.5–57.2)	0.92 (0.89–0.97)***
Full	29.2 (28.7–29.8)***	27.0 (26.4–27.6)***	54.4 (53.8–55.1)	0.86 (0.78–0.94)***
*Sex of the children*				
Male®	39.2 (38.9–39.5)	36.4 (36.2–36.7)	58.5 (58.2–58.8)	–
Female	38.1 (37.8–38.4)***	35.7 (35.4–36.0)***	58.8 (58.5–59.2)	1.18 (1.13–1.22)***
*Age of the children*				
1 year®	21.8 (21.4–22.2)	28.0 (27.6–28.5)	68.5 (67.9–69.2)	–
2 years	43.0 (42.5–43.5)***	35.4 (35.0–35.9)***	70.6 (70.2–71.0)***	–
3 years	43.0 (42.5–43.5)***	38.0 (37.6–38.5)***	62.4 (61.9–62.8)***	–
4 years	43.7 (43.2–44.1)***	38.7 (38.3–39.2)***	52.2 (51.8–52.7)***	–
5 years	40.3 (39.8–40.8)***	39.4 (39.0–39.9)***	44.7 (44.3–45.2)***	–
*Regions*				
Others®	32.0 (31.7–32.3)	30.7 (30.4–30.9)	55.9 (55.5–56.2)	–
EAG	39.7 (39.3–40.1)***	38.8 (38.4–39.2)**	57.8 (57.3–58.2)***	1.41 (1.34–1.49)***
Uttar Pradesh	46.6 (46.1–47.1)***	39.9 (39.4–40.4)***	63.4 (62.9–63.9)***	2.02 (1.90–2.15)***
Bihar	48.7 (48.1–49.3)***	44.2 (43.6–44.8)***	63.6 (63.0–64.1)***	1.13 (1.04–1.22)***
*Total*	38.7 (38.5–38.9)	36.1 (35.9–36.3)	58.7 (58.4–58.9)	–
*Number of observations*	2,23,011	2,23,011	2,07,594	5,07,265
*Log pseudo likelihood*	−132135	−131640	−127078	−142369
*Wald chi*^*2*^	9130.37***	7454.68***	7011.4***	4470.23***

CI, confidence interval; IBMFB, interval between marriage and first birth; IBBSB, interval between birth and subsequent birth; MCA, multiple classification analysis; EAG, Empowered Action Groups.

Estimates are weighted with national women weight; Cox proportional hazard ratios has been presented for under-five child mortality; upper and lower limit of confidence interval have been shown in the parentheses; *P < 0.10, **P < 0.05, ***P < 0.01. ® stands for reference category of the variable.

a Estimates based on logistic regression and MCA conversion model.

b Estimates based on Cox proportional hazard regression model.

### Child health outcomes

The association between child health outcomes, notably stunting, underweight, anemia, and under-five mortality, and the planning of births including other confounders is presented in Table 3. The results show that the possibility of stunting is significantly higher among the children born to women with two births and <3 years of IBBSB (41.1%, *P* < 0.01), three births and <3 years of IBBSB (46.8%, *P*<0.01), >3 births and <3 years of IBBSB (53.1%, *P* < 0.01), and >3 births and >3 years of IBBSB (47.3%, *P* < 0.01) than that of children born to women with one birth and >2 years of IBMFB (32.5%) and one birth and 2–3 years of IBMFB (33.0%, *P* < 0.10). The EAG states (39.7%, *P* < 0.01), Uttar Pradesh (46.6%, *P* < 0.01), and Bihar (48.7%, *P* < 0.01) had a considerably higher rate of stunting than that of the rest of India (32.0%). Excluding the occupational status of the mother, other socio-economic and demographic characteristics of the children were significantly correlated with their nutritional outcomes.

From Table 3, the results of childhood underweight suggests that the probability of being underweight is higher among children born to mothers with two births and <3 years of IBBSB (38.1%, *P* < 0.01), three births and <3 years of IBBSB (43.0%, *P* < 0.01), >3 births and <3 years of IBBSB (48.4%, *P* < 0.01), and >3 births and >3 years of IBBSB (43.5%, *P* < 0.01) than that of children born to mothers with one birth and <2 years of IBMFB (30.7%). The rate of being underweight was higher among children living in EAG states (38.8%, *P* < 0.05), Uttar Pradesh (39.9%, *P* < 0.01), and Bihar (44.2%, *P* < 0.01) compared with that of the other states of India. On the line of previous studies, the other socio-economic factors, barring the occupational statuses of mothers, were significantly correlated with childhood underweight.

The estimates showing the association between the anemia level of the children and the planning of births is presented in Table 3. The results suggest that the probability of being anemic was higher among the children born to mothers with one birth and <3 years of IBMFB (57.5%, *P* < 0.01), two births and <3 years of IBBSB (60.2%, *P* < 0.01), three births and <3 years of IBBSB (63.5%, *P* < 0.01), three births and >3 years of IBBSB (59.4%, *P*<0.01), >3 births and <3 years of IBBSB (64.9%, *P* < 0.01), and >3 births and >3 years of IBBSB (62.6%, *P* < 0.01) than that of children born to mothers with one birth and <2 years of IBBSB (54.1%), two births and >3 years of IBBSB (55.8%, *P* < 0.01), and one birth and 1–2 years of IBMFB (56.1%, *P* < 0.10). The EAG states (57.8%, *P* < 0.01) have lower prevalence, while Uttar Pradesh (63.4%, *P* < 0.01) and Bihar (63.6%, *P* < 0.01) have higher prevalence of anemia among the children than that of the other states of India (55.9%). Child anemia was also significantly associated with other socio-economic and demographic characteristics, except for mother's occupational status and exposure to mass media, the father's educational level, and the sex of the child. However, in case of child anemia, the planning of births emerges as the most significant predictor, showing greater differences in the anemia levels compared with that of any of the socio-economic indicators.

**Fig. 1:**
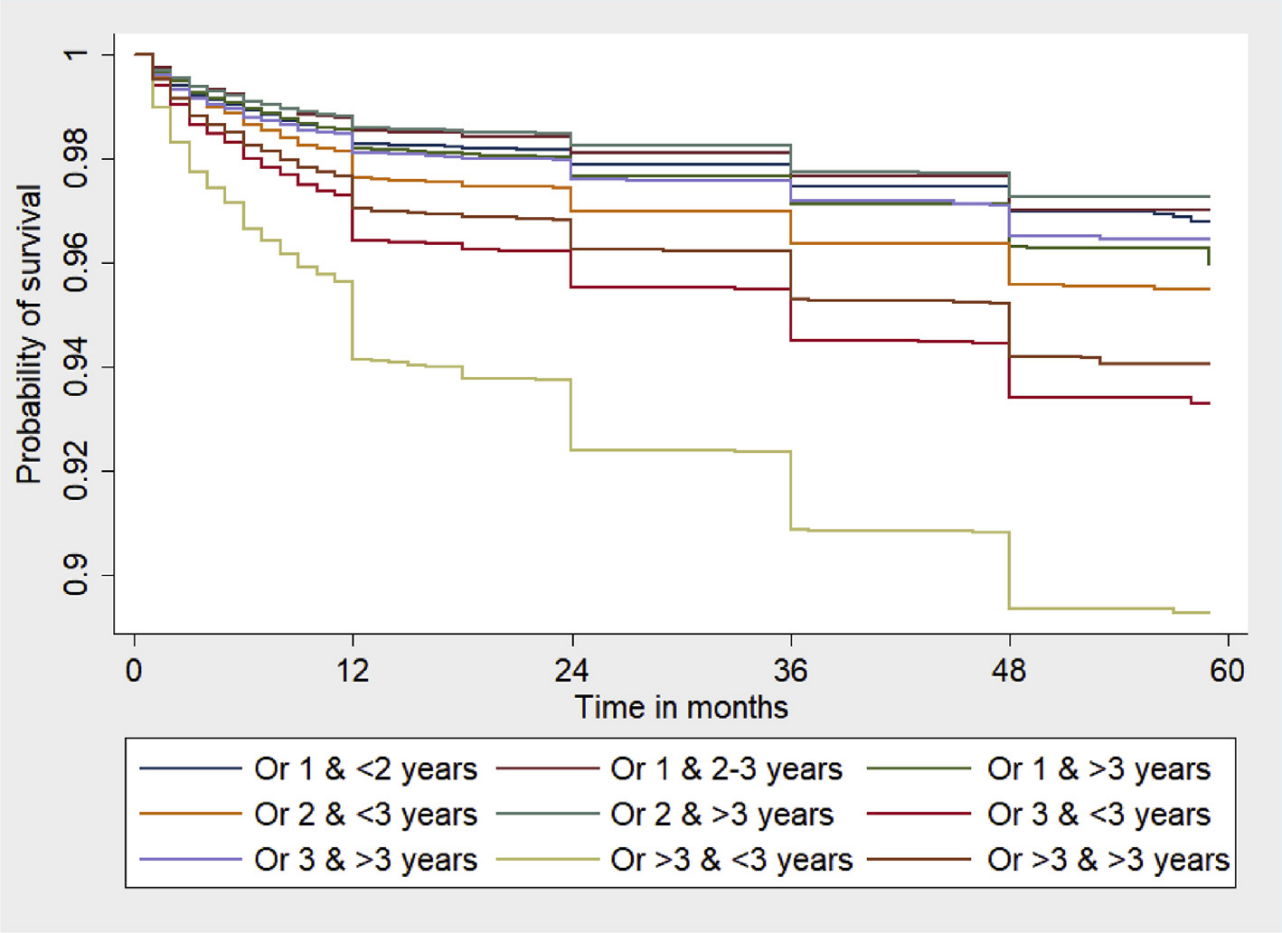
Kaplan–Meier survival estimates of under-five children in India, 2015–16. Or 1 & <2 years = Order 1 & <2 years of IBMFB; Or 1 & 2–3 years = Order 1 & 2–3 Years of IBMFB; Or 1 & >3 years = Order 1 & >3 Years of IBMFB; Or 2 & <3 years = Order 2 & <3 Years of IBBSB; Or 2 & >3 years = Order 2 & >3 Years of IBBSB; Or 3 & <3 years = Order 3 & <3 Years of IBBSB; Or 3 & >3 years = Order 3 & >3 Years of IBBSB; Or >3 & <3 years = Order >3 & <3 Years of IBBSB; Or >3 & >3 years = Order >3 & >3 Years of IBBSB. IBMFB, interval between marriage and first birth; IBBSB, interval between birth and subsequent birth.

The Kaplan–Meier estimates of survival during the childhood are displayed in Fig. 1. The curves suggest that the probability of dying is highest among the children of mothers with >3 births and <3 years of IBBSB, followed by those with three births and <3 years of birth spacing, >3 births and <3 years of birth spacing, and two births and <3 years of birth spacing. There was not much variation in the probability of dying observed among the rest of the groups.

The adjusted hazard ratios from the Cox proportional hazard regression analysis showing the association between under-five mortality and the planning of births after controlling for other socio-economic and demographic characteristics are presented in Table 3. The results demonstrate that the risk of death is lower among children born to mothers with one birth and 2–3 years of IBMFB (hazard ratio [HR] 0.81, *P* < 0.01), one birth and >3 years of IBMFB (HR 0.88, *P* < 0.01), two births and >3 years of IBBSB (HR 0.61, *P* < 0.01), three births and <3 years of IBBSB (HR 0.62, *P*<0.01), and >3 births and >3 years of IBBSB (HR 0.72, *P* < 0.01) compared with that for children born to mothers with one birth and >2 years of IBMFB. Compared to the children born to mothers with one birth and >2 years of IBMFB, the risk of child death was higher among those with three births and <3 years of IBBSB (HR 1.11, *P*<0.01) and >3 births and <3 years of IBBSB (HR 1.37, *P* < 0.01). The hazard of childhood death was higher in the EAG states (HR 1.41, *P* < 0.01), Uttar Pradesh (HR 2.02, *P* < 0.01), and Bihar (HR 1.13, *P* < 0.01) than that in the rest of the country. Except the place of residence, other socio-economic and demographic factors were significantly correlated with the under-five mortality.

## Discussion

This study provides a comprehensive assessment of the effects of the planning of births (by adopting family planning) on select key maternal, child health, and nutritional outcomes using the most recent national family health survey data. The findings suggest that the selected maternal, child health, and nutritional outcomes (viz. BMI and anemia level of women as well as the stunting, underweight, anemia, and mortality of children) significantly differed by the intersectional axes of the planning of births. In particular, the risk of childhood underweight is considerably higher among children born to women who have had three or more births and less than 3 years of birth spacing compared with risk for those with one birth and more than 2 years of spacing between marriage and their first birth. Similarly, among the women with more than one birth and shorter birth spacing, the likelihood of being anemic is higher than that among those with one birth and greater than 2 years of spacing between marriage and the first birth. Compared to the children with mothers who have longer birth spacing and a lower number of births, the likelihood of stunting, underweight, and anemia is considerably higher among those born to women with more than one birth and less than 3 years of birth spacing. The hazard of child death is substantially higher among those with three births or more and less than 3 years between births, while it is lower among those with one birth and more than 2 years between births, as well as those with more than two births and more than 3 years between births. Thus, this study has found that the planning of births is significantly associated with maternal, child health outcomes.

The findings from the present study support the arguments put forward by the previous studies in global and South Asian contexts.^3^, ^5^, ^14^, ^16^, ^18^ In particular, early marriage, early childbearing, and lack of family planning lead to a shorter time between marriage and a first birth, which results in poor nutritional outcomes of the women.^16^, ^18^ Furthermore, women with more births and shorter birth spacing have a greater risk of being underweight than their counterparts do.^3^ This study shows that the risk of anemia among women and children is higher for a greater number of births, while previous studies have suggested that the direction of the relationship between birth spacing and maternal and child anemia is not just one way; instead, the direction varies from positively significant to not significant.^14^ In the present study, among the women and their children from their first birth, the risk of being anemic was higher among those with more than 3 years of spacing between marriage and the birth than that among those with a gap of less than 2 years. Further research is needed for this contradictory findings. The childhood nutritional outcomes, notably stunting and underweight, were also associated with the planning of births for timing, spacing, and limiting, which is constituent with that reported previously.^4^ Similar to previous studies,^29^, ^30^ this study's findings also suggest that the children born to mothers with a greater number of births with shorter birth spacing have a greater risk of under-five death compared with the risk for those born to mothers as their first birth.

The findings of this study are based on a more nuanced assessment of a larger number of indicators and re-strengthen the argument that the intersectional axes of the planning of births comprising the timing, spacing, and limiting of births have a biodemographic relationship with maternal and child health outcomes. The adolescent period of women is an important period of physical growth and development.^5^ Pregnancy and lactation during this period leads not only to the depletion of nutritional elements (fat, iron, and folate) among them but also in their children. This depletion of fat and iron among the mothers causes underweight and anemia among them and poor pregnancy and delivery outcomes among their children. These poor outcomes drive the children to be underweight, stunted, and anemic, which in turn results in childhood mortality. In similar findings, shorter birth spacing and repeated pregnancy and delivery (higher number of births) hinder women in recovering their body weights and other micronutrients. As a result, these women are more likely to experience poor pregnancy and delivery outcomes such as IUGR, LBW, premature birth, and small birth size, which are risk factors for adverse childhood growth and higher mortality.

As mentioned earlier, the pathways through which family planning affect maternal, newborn, and child health and nutrition are both direct and indirect. By helping couples attain the number of children they want at the healthiest times in their lives, family planning can benefit mothers, infants, and children. The adequate spacing of births allows women's bodies to recover and restock vital nutrients and leads to better maternal, newborn, and child health and nutritional outcomes, such as healthy pregnancy outcomes and lower childhood mortality. Family planning can help adolescents to delay pregnancy until an ideal reproductive age (>18 years) and thus can improve their growth and development and reduce the risk of poor nutritional and health outcomes for their infants. A growing body of evidence shows that intentional pregnancy can also influence nutritional outcomes.^4^, ^6^, ^7^, ^9^ The children from unintended pregnancies have a higher risk of poor nutrition, underscoring the important role of family planning.^10^, ^21^

Family planning indirectly affects nutrition via its impact on infant and young child feeding practices. When births are well spaced, mothers have more time, energy, and resources to adequately breastfeed and feed their young infants and children, respectively. Research shows that when pregnancies are planned and occur when women are older than 18 years, breastfeeding practices improve, leading to improved nutrition of the infants.^2^, ^3^, ^4^, ^11^ When unplanned pregnancies are avoided, women are less exposed to the risks of dying due to pregnancy and childbirth. Since mothers play a crucial role in feeding their families, reductions in maternal death can positively influence infant and child nutrition. Finally, family planning can have an indirect impact on nutrition by reducing unintended pregnancies among adolescents, allowing them to stay in school and complete more years of education. Research shows that greater education among women leads to greater productivity, empowerment, and control of resources—allowing them to make better choices that ultimately benefit both them and their children's health and nutrition.^4^, ^6^, ^7^, ^8^, ^9^, ^10^

This study has some limitations and strengths that must be noted. We used cross-sectional data to draw the underlying association between the planning of births and maternal and child health outcomes, but for establishing a perfect causal relationship, a longitudinal design of experiments is required. As the purpose of this study is to link family planning to maternal, newborn, and child health and nutritional outcomes, both past and current use of contraception are not appropriate for establishing this relationship. However, by constructing a proxy variable for representing family planning use in the form of the planning of births (the continuum process of timing, spacing, and limiting), this study fills a critical gap by providing timely empirical evidence on linking family planning to maternal, newborn, and child health and nutritional outcomes. The spacing between marriage and first birth was included in the planning of births variable, which is often overlooked in most studies on the process of linking family planning to maternal, newborn, and child health and nutritional outcomes, and it emerged as one of the essential biodemographic factors of the latter. This study advances the strengthening of strategies of integration of family planning with maternal, newborn, and child health and nutrition in India.

### Conclusions

This study provides several cross-cutting implications for clinical practice and health policymaking. The findings from this study show that the planning of births has a biodemographic advantage in improving maternal and child health outcomes. Given the evidence that more than one-fourth of all adolescent girls experience child marriages in India,^2^ a longer spacing between marriage and the first birth could improve the maternal, child health outcomes, notably, underweight of women and stunting, underweight, and mortality of children. Family planning programs in India have always been heavily skewed toward limiting methods, especially female sterilization, since it is cost effective for the policymakers to control population growth,^30^ but appropriate spacing between births also has an important effect on better maternal and child health outcomes. Apart from limiting, spacing methods of family planning must be emphasized for better health outcomes. Fertility declines have almost reached the replacement level in the country despite a lower level of contraceptive use and a high unmet demand for family planning;^31^ this does not mean in any way that the relevance of family planning can be underestimated. The high unmet demand for family planning in both spacing and limiting must be addressed not only to meet population stabilization goals but also to accomplish the SDG goals of health for all. Although the FP2020 vision document of the Ministry of Health and Family Welfare aimed to reach an additional 48 million women and girls with family planning methods,^32^ the findings from this study encourage the strategy of universal coverage of contraceptive use in India for achieving the holistic benefits of family planning, such as better maternal, newborn, and child health and better nutritional outcomes.

The integration of family planning, maternal, newborn, and child health and nutritional programs has multiple opportunities to provide family planning counseling and services to the mothers during their postnatal care at the health centers. This integration is also less time consuming and is cost effective for the healthcare system; it also helps in improving birth spacing and avoids unintended births. The evidence shows that a few countries have harvested these opportunities for providing family planning counseling and services to this ‘captive audience’.^33^ In India, to a great extent, the integration of family planning with maternal, newborn, and child health and nutrition has not succeeded to the extent that it was targeted under successive population policies due to lack of a true integration strategy at the implementation level, service delivery at the peripheral level, a shortage of frontline health workers, and consequent overburdening of them.^34^ United Nations has defined linkages as ‘policy, programatic, services, and advocacy of bidirectional synergies’ between maternal, newborn, and child health and nutrition and family planning.^35^ In contrast to linkages, which exist at multiple levels, these organizations define integration at the service delivery level only as ‘different kinds of services or operational programs joined together to ensure and perhaps maximize collective outcomes.’ Therefore, India needs to revise their integration mechanism and eliminate disconnects that hinder the delivery of these services.

## Author statements

### Ethical approval

This study used publicly available secondary sources of data. Thus, it does not require ethical approval.

### Funding

This study was funded by the Bill & Melinda Gates Foundation, India Country Office, New Delhi, India. Grant Number: OPP1142874.

### Competing interest

The authors have no conflicts of interest to declare.
